# A Stochastic Description of *Dictyostelium* Chemotaxis

**DOI:** 10.1371/journal.pone.0037213

**Published:** 2012-05-25

**Authors:** Gabriel Amselem, Matthias Theves, Albert Bae, Eberhard Bodenschatz, Carsten Beta

**Affiliations:** 1 Max Planck Institute for Dynamics and Self-Organization, Göttingen, Germany; 2 School of Engineering and Applied Sciences, Harvard University, Cambridge, Massachusetts, United States of America; 3 Institute of Physics and Astronomy, University of Potsdam, Potsdam, Germany; 4 Laboratory of Atomic and Solid State Physics, Cornell University, Ithaca, New York, United States of America; 5 Institute for Nonlinear Dynamics, University of Göttingen, Göttingen, Germany; Cardiff University, United Kingdom

## Abstract

Chemotaxis, the directed motion of a cell toward a chemical source, plays a key role in many essential biological processes. Here, we derive a statistical model that quantitatively describes the chemotactic motion of eukaryotic cells in a chemical gradient. Our model is based on observations of the chemotactic motion of the social ameba *Dictyostelium discoideum*, a model organism for eukaryotic chemotaxis. A large number of cell trajectories in stationary, linear chemoattractant gradients is measured, using microfluidic tools in combination with automated cell tracking. We describe the directional motion as the interplay between deterministic and stochastic contributions based on a Langevin equation. The functional form of this equation is directly extracted from experimental data by angle-resolved conditional averages. It contains quadratic deterministic damping and multiplicative noise. In the presence of an external gradient, the deterministic part shows a clear angular dependence that takes the form of a force pointing in gradient direction. With increasing gradient steepness, this force passes through a maximum that coincides with maxima in both speed and directionality of the cells. The stochastic part, on the other hand, does not depend on the orientation of the directional cue and remains independent of the gradient magnitude. Numerical simulations of our probabilistic model yield quantitative agreement with the experimental distribution functions. Thus our model captures well the dynamics of chemotactic cells and can serve to quantify differences and similarities of different chemotactic eukaryotes. Finally, on the basis of our model, we can characterize the heterogeneity within a population of chemotactic cells.

## Introduction

Directional movement of cells in response to chemical cues is ubiquitous in nature. It is essential for many biological processes ranging from embryogenesis [1], to wound healing [2] and cancer metastasis [3]. A complete picture of how a eukaryotic cell senses, responds, and migrates in a chemical gradient is still missing. This includes the many unknown molecular details in the chemotactic signaling pathway [4], [5] as well as the lack of a quantitative model to describe the chemotactic process. Many approaches have been developed to advance our quantitative understanding of eukaryotic chemotaxis. Among them, the use of GFP-tagged constructs and knock-out mutants has emerged as the most prominent tool to assess the role of individual proteins in the chemotactic process.

The chemotactic performance of a cell line is commonly investigated using gradient methods like micropipette assays or diffusion chambers. To date, chemotaxis in such assays has been characterized based on averaged quantities taken over a population of cells as well as over time. Typical examples are the average velocity in gradient direction [6] or the chemotactic index, a measure of the average angle of propagation relative to the gradient direction [7]. Such measures show a deterministic dependence on the gradient signal. Nevertheless, they only convey very limited information about the chemotactic movement. In particular, they do not reflect the fluctuations that are inherent in all dynamical processes at the cell level. This element of randomness is a salient feature of cell movement that may vary strongly between different mutant cell lines and requires detailed quantification. Recently, a model was proposed that describes the chemotactic motion of a cell as a stochastic process governed by the probabilities of pseudopod extension [8]. These probabilities have been determined from experiments, including the gradient induced bias in the case of chemotactic motion. Based on this model, Monte Carlo simulations were performed and chemotactic indices computed from the resulting random walks. A close agreement with experimental data was found on the level of the chemotactic index. The chemotactic index, however, is a global measure of the average direction of cell motion. A more refined description of eukaryotic chemotaxis should also take into account the fluctuations of the cell velocity observed in experimental data. It is the aim of this article to develope a statistical description of eukaryotic chemotaxis that captures these details quantitatively and serves as a benchmark to describe eukaryotic chemotaxis.

Processes that exhibit both deterministic and stochastic components are commonly described by Langevin-type stochastic differential equations. This approach has a long-standing tradition in the study of random (non-chemotactic) cell motion. The first random walk models that describe the motion of microorganisms date back to the early 20th century [9], [10]. In the 1990s, Langevin equations were introduced for the first time to describe cellular motion, see Ref. [11] among others. Such models have been adapted to various organisms. They were extended to include a chemotactic bias [12], [13] as well as coupling between speed and turning angle [14]. Recently, a systematic, model-free analysis was proposed to extract the parameters of a Langevin equation directly from experimental data without *a priori* modeling assumptions [15]. Since then, similar statistical approaches have been adopted by various groups to describe random ameboid motion in absence of external cues [16]–[19]. They have also stimulated interests among theoretical scientists that study generic models of actively moving particles [20], [21].

Here, we introduce an analogous statistical concept to describe the directional movement of chemotactic cells in a chemical gradient. Earlier Langevin-type chemotaxis models were based on the assumption that random cell motion can be described as an Ornstein-Uhlenbeck process [12], [13]. However, recent work has shown that this is not necessarily the case [15], [17], [19]. Inspired by these recent studies of random cell migration, we also base our present analysis on a generalized Langevin equation,
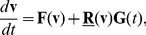
(1)where 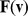 is the deterministic component of cell motion and 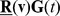 represents the random contributions with 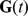 denoting Gaussian white noise. The functions 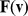 and 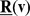 can be determined directly from experimental data by conditional averaging. In the presence of a chemical gradient however, both the deterministic and the stochastic parts may depend on direction. Thus, conditional averages have to be taken in an angle-dependent fashion, requiring a much higher data density as compared to the analysis of random motion. To obtain large numbers of cell tracks under well-controlled conditions, we employed microfluidic devices as our experimental platform [22]. The experiments were performed with chemotactic cells of the social amoeba *Dictyostelium discoideum*, a common model organism for cell motility and chemotaxis [23]. From the cell tracks, we determined the deterministic and stochastic parts of our model equation 1. This analysis was systematically performed for cell populations experiencing different gradients of chemoattractant. We furthermore divided the cells into several subpopulations according to their speed and directionality. The same formalism was then applied to each subpopulation to exemplify the relation between the model parameters and different modes of cell movement.

In summary, it is the overall objective of our work to advance our understanding of eukaryotic chemotaxis beyond a description in terms of averaged values. In particular, we will characterize the deterministic and stochastic components of chemotactic motion along with their dependence on external parameters. Our primary goal is thus to phrase a detailed statistical description of chemotactic motion that captures also the distribution functions of fluctuating quantities. At this stage, it remains a purely descriptive approach. In future studies, it will serve as a basis for the detailed comparison of different mutant cell lines. This will enable us to identify the molecular players that determine specific features in the motion patterns of eukaryotic cells and link our model parameters to the underlying signaling events. Ultimately, this will lead to a detailed understanding of how eukaryotic cells move in response to a chemical gradient, a long-term aim of quantitative biology.

## Results

### Microfluidic Tools Allow Quantitative Recording of Large Chemotaxis Data Sets

**Figure 1:**
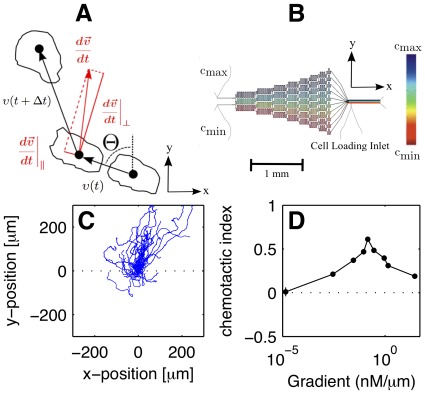
Experimental setup. (A) Definition of the coordinate system. (B) Microfluidic gradient mixer, adapted from [6]. The x-direction of the coordinate system corresponds to the direction of fluid flow in the main channel of the device, the y-direction to the direction of the chemoattractant gradient. (C) Trajectories of chemotactic *Dictyostelium* cells in a gradient of 0.16 nM/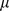
m cAMP. The starting point of all trajectories was shifted to (0,0). (D) Average chemotactic index as a function of the cAMP gradient. Note that the data point displayed at very low gradient values (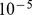
nM/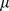
m) corresponds to an experiment where no gradient of cAMP was applied.

We studied the chemotactic motion of starvation developed *D. discoideum* cells in stable linear gradients of cyclic AMP (cAMP). The gradients were generated using a pyramidal microfluidic network that provides well-defined concentration profiles with high temporal stability. The layout of our gradient device can be seen in Fig. 1B. It is a modified version of the classical design introduced by Jeon and coworkers [24]. The device has been thoroughly characterized and was successfully used in previous studies of *D. discoideum* chemotaxis, for details see Ref. [6].

In our experiments, cAMP gradients were linearly extending over a distance of about 320
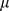
m inside the microfluidic chamber, ranging from zero on one side to a maximal concentration level 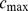 on the other side. The value of 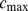 was systematically varied between different experiments, to cover the entire range of gradients over which *D. discoideum* shows directional responses [6]. Compared to our earlier work, we collected much larger data sets in order to evaluate the parameters of our model in an angle-resolved fashion. In Fig. 1C, cell tracks recorded in a cAMP gradient of 0.16 nM/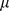
m are displayed as an example. At each time, the velocity of the cell was determined by finite differences. From the velocity, we calculated the chemotactic index of each cell according to 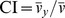, where 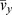 is the average velocity of the cell in gradient direction, and 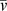 is the average cell speed. This corresponds to the ratio between the distance travelled in the gradient direction and the total length of the trajectory. In Fig. 1D, the chemotactic index is displayed as a function of gradient steepness. Note that the data point displayed at very low gradient values (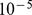
nM/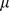
m) corresponds to an experiment where no gradient of cAMP was applied. In agreement with our earlier work, we observed an optimal chemotactic performance around 0.1 nM/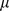
m [6].

**Figure 2:**
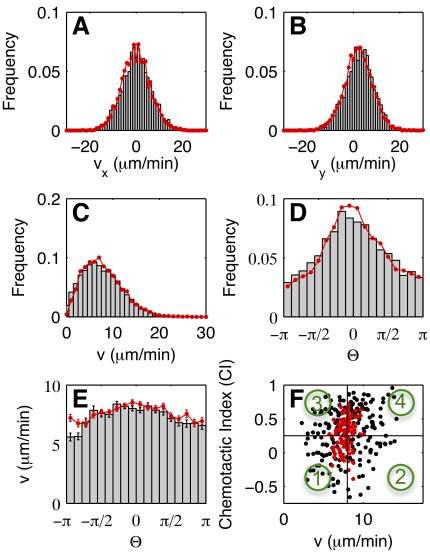
Comparison of experimental and simulated histograms. Experimental histograms (gray boxes) and simulated histograms (red lines) of (A) 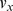, (B) 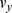, (C) 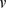, and (D) 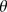. (E) Experimental (gray boxes) and numerical (red line) distributions of 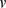 as a function of 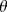. (F) Each dot marks a cells according to its mean speed and chemotactic index in the (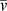,CI)-plane. Black symbols mark the experimental data, red dots the numerical results. The vertical and horizontal lines indicate the mean speed and chemotactic index of the entire population as obtained from the experiment. The numbers mark the subpopulations defined by the four quadrants. They are differentiated according to their directionality and speed, (1) slow non-chemotactic, (2) fast non-chemotactic, (3) slow chemotactic, and (4) fast chemotactic cells.

After considering the chemotactic index as a classical average measure of the chemotactic performance, we moved on to analyze the fluctuations in various motion parameters by extracting the probability distribution functions of these quantities from the data. The results are summarized in Fig. 2, where the experimental data is displayed in gray bars and black dots. Along with the experimental data, numerical simuations based on the model equations (2) and (3) are shown in red. The simulations will be discussed at a later point in the Results Section, after the model equations have been introduced. In Fig. 2, the probability distribution functions (PDF) for the velocity components (A and B), the speed (C), and the propagation angle (D) over the entire population are shown. Furthermore, the average speed depending on the angle of propagation was extracted from the data (E) and the relation between cell speed and chemotactic index (CI) was investigated in the form of a scatter plot in the (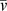,CI)-plane (F). In Fig. 2, these results are displayed for a gradient of 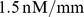 as an example. While the component perpendicular to the cAMP gradient (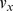) was distributed symmetrically around zero, the distribution of the component in gradient direction (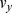) was shifted towards positive values, clearly reflecting the directional nature of the movement (see Fig. 1A for a definition of the coordinate frame). We furthermore observed that both the distributions of propagation angle and speed are peaked in gradient direction, see Fig. 2D and E. A weak correlation between the speed and the chemotactic index of the cells was found. To test for correlations, we marked each cell according to its average speed (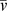) and its chemotactic index (CI) in the (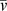,CI)-plane, see Fig. 2F. A correlation coefficient of 0.23 was found for this data set. This indicates that within a population of chemotactic cells, the more chemotactic ones tend to be more mobile, i.e., have a larger speed. This is in agreement with earlier studies, where it was reported that the motility of *Dictyostelium* cells increases over the first hours of development [25], so that the higher developed, and thus more chemotactic cells should also display a higher motility.

### The Deterministic Part of Directed Motion Depends on Gradient Direction while the Stochastic Part does not

It is our aim to model chemotactic motion based on the generalized Langevin equation 1. To phrase a specific model equation of this type, we need the explicit functional dependencies of the deterministic and stochastic parts on the cell speed and direction. We determined these expressions from our experimental data by conditional averaging [26], [27]. To retrieve the deterministic part, we divided the range of cell speeds into 20 intervals of equal size. In the same way, the range of propagation angles was divided into 18 equally sized intervals. We then averaged the acceleration of the cells within each interval to obtain the deterministic part as a function of speed and angle. Similarly, the stochastic part was determined by taking the variance of the fluctuations in acceleration for fixed speed and angle. For details of the conditional averaging procedure, see the Materials and Methods Section. Inspired by earlier work on Langevin models of random cell motion [15], we decomposed the acceleration into its projections parallel and perpendicular to the cell’s instantaneous velocity, see Fig. 1A.

**Figure 3:**
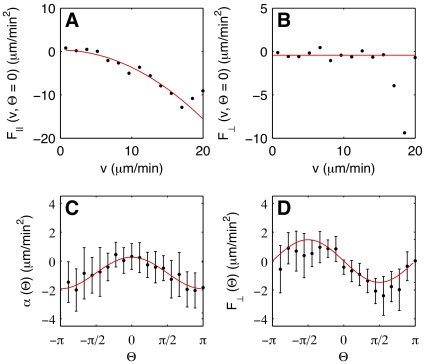
Deterministic components of the Langevin equation. Deterministic components of (A) the parallel and (B) the perpendicular acceleration for 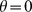 (gradient direction), as a function of 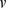. Black dots show the experimental results, the red lines display fits according to 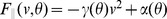 and 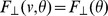, respectively. (C) 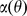 as a function of 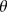. The red line shows the fit 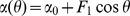. (D) 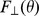 as a function of 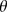. The red line shows the fit 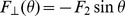. Error bars indicate the 95% confidence interval on the values of 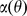 and 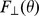.

Let us first consider the deterministic and stochastic parts in a fixed cAMP gradient of 1.5 nM/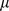
m. We found that the deterministic part of the acceleration parallel to the current direction of motion depended on both the speed (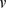) and the angle (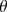) of propagation. It was well approximated by a quadratic fit, 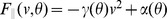. In Fig. 3A, we show 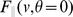, the deterministic part in gradient direction, as an example. The friction coefficient 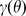 was found to be independent of 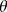 within the precision of our experiments, see Fig. S1 of the electronic supplementary material. The angular dependence of 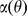 can be seen in Fig. 3C, together with a sinusoidal fit 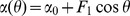 (see the equation for 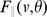 above for a definition of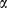). By contrast, the deterministic part perpendicular to the direction of motion was independent of the cell’s speed and dependent only on angle, 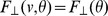. In Fig. 3B, 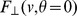 can be seen as an example. The angular dependence of 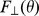 was well approximated by 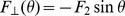, see Fig. 3D. In Fig. S2 of the electronic supplementary material, we show that 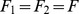. Thus, the presence of a gradient was reflected by an additional effective force in the deterministic part. It consisted of a contribution 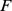, pointing along the gradient direction, and a contribution 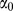 pointing along the direction of propagation.

**Figure 4:**
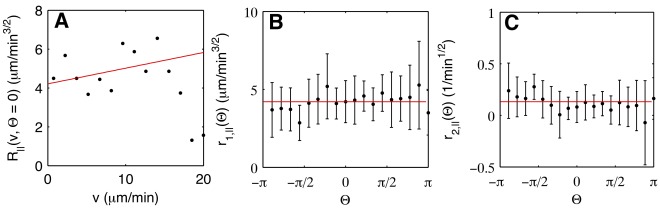
Stochastic components of the Langevin equation. (A) Stochastic component of parallel acceleration. Black dots show the experimental data, the red line shows a linear fit 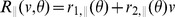. (B, C) 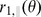 and 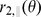 are independent of 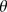. The red lines show constant fits.

For the stochastic part, we found multiplicative noise that can be approximated by a linear dependence on the cell speed. The noise parallel to the direction of motion could be fitted, at each angle, by a first-order polynomial 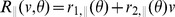, see Fig. 4A (see Fig. S7A and B of the electronic supplementary material for more examples of this curve at other values of 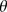). In Figs. 4B and C, the offset 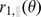 and the slope 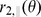 are shown as a function of the angle. No dependence on 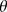 was observed. Similarly, the noise perpendicular to the direction of motion could be fitted by a first-order polynomial with angle-independent offset and slope, see Fig. S3 of the electronic supplementary material. Because no angular dependence was found in either of the stochastic components, we averaged the data over all angular bins and fitted the result by first order polynomials, 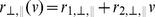. The stochastic part as a function of cell speed, averaged over all angles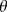, can be seen in Fig. S7C of the electronic supplementary material.

Thus, by conditional averaging, the following Langevin equation for the chemotactic movement of *D. discoideum* was obtained,
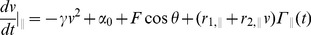
(2)

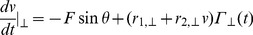
(3)


The model incorporates quadratic damping and multiplicative noise.

### The External Gradient Sets the Effective Force Terms 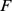 and 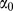


**Figure 5:**
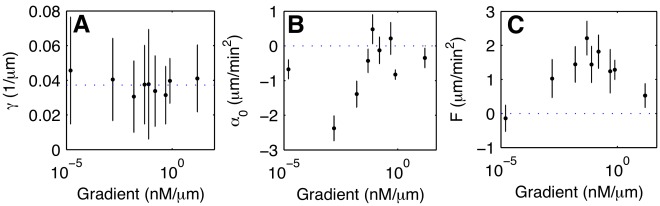
Evolution of the deterministic components with the gradient strength. (A) Friction coefficient 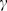, and effective force terms (B) 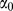, and (C) 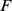 as a function of the gradient. The error bars indicate the standard deviation in (A) and the 95% confidence intervals in (B) and (C). As in Fig. 1D, the data point displayed at very low gradient values (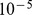
nM/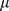
m) corresponds to an experiment where no gradient of cAMP was applied.

In the previous section, we have derived a probabilistic model of chemotactic motion in one given gradient of 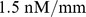. How do the model parameters depend on the steepness of the chemoattractant gradient? To answer this question, we repeated the above analysis for chemotactic motion in gradients ranging over four orders of magnitude, see Fig. 1D. The results are summarized in Fig. 5, where the friction coefficient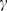, as well as the parameters 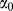 and 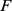 are displayed as a function of gradient steepness. While 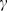 did not show any dependence on the gradient, both 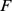 and 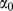 went through a maximum at about 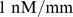. This coincided with a peak in the chemotactic index as shown in Fig. 1D, and with a peak in the motility [6]. For the stochastic components, no clear dependence on the gradient steepness was observed, see Fig. S4 of the electronic supplementary material. Thus, the effect of a chemoattractant gradient on chemotactic cell motion was encoded in the effective force terms 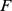 and 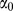. All other model parameters did not show any gradient dependence and are constitutive properties of the cell.

### Velocity and Angle Distributions of the Population are Captured by the Langevin Model While Cellular Individuality is not

We used an Euler-Maruyama scheme to simulate the model equations 2 and 3 based on the parameters that were retrieved from the experimental data. For details of the numerical scheme see Materials and Methods. In Fig. 2A-E, the velocity histograms, the distribution of the propagation angle, and the dependence of the average speed on the propagation angle are displayed for a gradient of 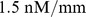. Together with the experimental data, the results of our numerical simulations are shown. We found close agreement between experiments and simulation.

In Fig 2F, the average speed (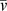) and the chemotactic index (CI) of each simulated cell track are marked in the (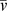,CI)-plane and compared with the experimental data. We observed that the scatter in the experimental data is greater than in the numerical simulations. The reason for this difference is that the model parameters were computed based on conditional averages of the entire population. Subsequent model simulations of cell tracks were all based on this set of averaged parameters. Thus, our model correctly recovered the chemotactic behavior (including probability distribution functions) at the population level, but not at the level of individual cell tracks.

### Different Modes of Chemotactic Motion are Reflected in Distinct Parameters of the Langevin Equation

**Figure 6:**
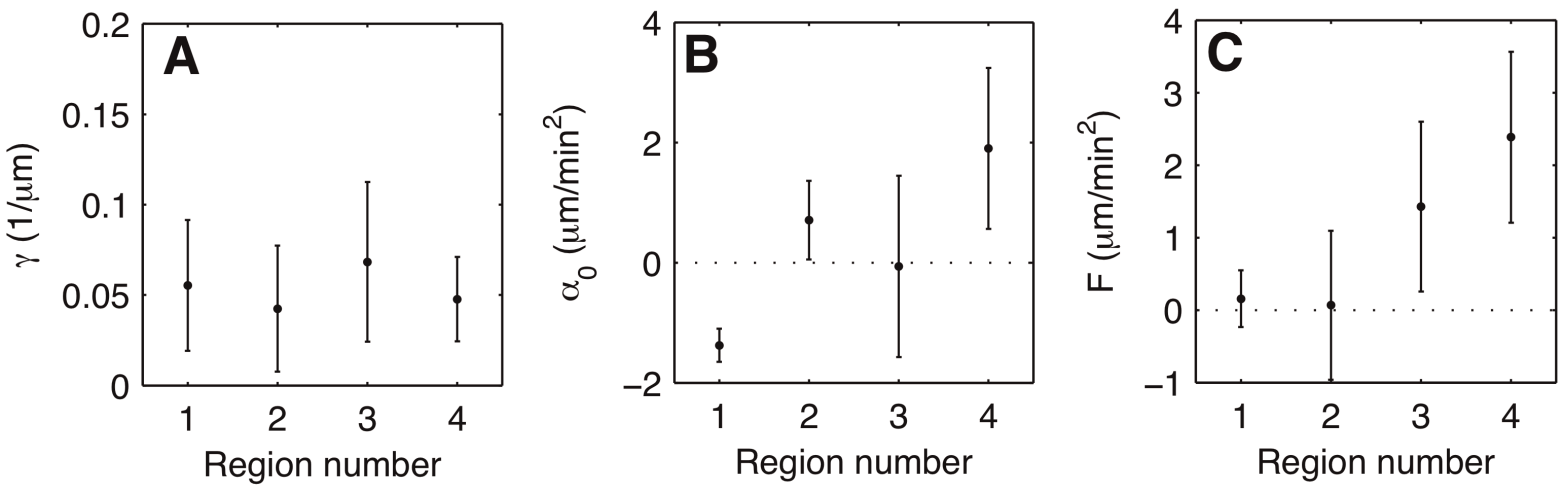
Evolution of the deterministic components at a given gradient, for each subpopulations. (A) Friction coefficient 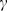, and effective force terms (B) 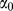, and (C) 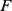 for each subpopulation. The error bars indicate the standard deviation in (A) and the 95% confidence intervals in (B) and (C).

To illustrate how the model parameters reflect different types of cell motion within a population, we have divided the data set of Fig. 2 into four subpopulations according to directionality and speed, (1) slow non-chemotactic, (2) fast non-chemotactic, (3) slow chemotactic, and (4) fast chemotactic cells. The numbers correspond to the quadrant labels in the (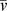,CI)-plane displayed in Fig. 2F. The lines along which the population was divided into the four quadrants, are chosen to coincide with the average cell speed and chemotactic index. There is no further intrinsic criterion for a separation into subpopulations. For each of these subpopulations we derived the model equations 2 and 3. The friction coefficient 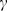 and the effective force terms 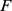 and 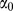 are shown in Fig. 6 for all subpopulations. While the friction coefficient showed only slight variations between the subpopulations, 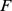
and 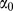 exhibited a clear trend. There was a positive 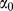 for subpopulations (2) and (4), reflecting their large mean speed. The force term 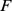 showed large positive values for (3) and (4), which corresponded to the high chemotactic index of these subpopulations. Also the parameters of the stochastic part showed variations between the four subpopulations, see Fig. S5 of the electronic supplementary material. The clearest trend was observed for the offset parameters 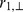 and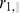. They were larger for the subpopulations (2) and (4) as compared to (1) and (3). Thus, the level of noise increased with increasing cell speed, irrespective of the chemotactic behavior of the cell.

## Discussion

**Figure 7:**
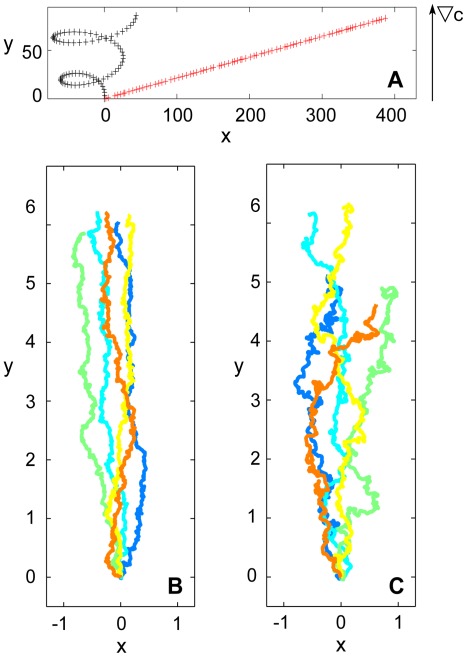
Schematic trajectories. (A) Two examples of schematic trajectories are displayed that have the same chemotactic index and the same average speed, but a very different geometrical character. (B) Trajectories governed by the Langevin equation 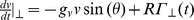, 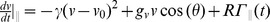. (C) Trajectories of an Ornstein-Uhlenbeck process with drift, 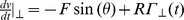, 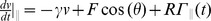. Also the trajectories in (B) and (C) have the same chemotactic index and the same average speed. The numbers on the axes are arbitrary space units.

We have recorded large data sets of *Dictyostelium* chemotaxis in linear gradients of cAMP using microfluidic tools. Different steepnesses were systematically explored, covering the full range of gradients, in which chemotactic behavior was observed [6]. Based on this data set, we derived a probabilistic model of eukaryotic chemotaxis. What is the benefit of this stochastic description? To date, chemotaxis is almost exclusively described by averaged quantities, most prominently, the average cell speed and the chemotactic index, which is defined as the distance moved in gradient direction divided by the total distance moved. However, cell trajectories with the same chemotactic index and the same average speed can be of very different type. To illustrate this, we show in Fig. 7A two schematic trajectories that have the same chemotactic index but very different geometrical character. In other more realistic cases, it may be difficult to judge the difference between two trajectories by eye even though their shape may be determined by very different underlying processes. As an example, we show in Figs. 7B and C trajectories that were generated by two different Langevin-type equations (see the caption of Fig. 7 for the form of these equations). They were designed to have the same chemotactic index and the same average speed. The differences between these trajectories can be only captured by carrying out the stochastic data analysis proposed here.

Thus, when considering only the chemotactic index, many details of the cellular motion patterns are lost. For example, when comparing mutant cell lines with deficiencies in different cytoskeletal regulators, the character of the cell trajectories may change considerably without substantial changes in the chemotactic index. Such differences in the structure of the cell trajectories may yield interesting information about the role of the respective proteins in the regulatory network of the cytoskeleton and cannot be resolved by the chemotactic index alone.

Here, our stochastic model of chemotactic cell motion will make a contribution. Using this more detailed description, it is possible to capture subtleties that go far beyond the information that is contained in the chemotactic index. We based our model on the assumption that chemotactic cell motion contains both deterministic and stochastic contributions. Such processes can be typically described by a Langevin-type equation. By applying angle-resolved conditional averaging to the experimental data, we obtained the deterministic and stochastic parts of the underlying Langevin equation and analyzed the dependence on the external gradient. To date, similar data-driven stochastic modeling has been only applied to non-directional, random cell motion in absence of external stimuli, see e.g. Refs. [15], [16], [18], [19]. In the present work, we have generalized this approach for the first time to describe the directed migration of eukaryotic cells in an external gradient of chemoattractant. In particular, we made the following observations:

- The stochastic equation of motion showed quadratic damping and multiplicative noise, similar to non-directional random ameboid motion (see Fig. S6 of the electronic supplementary material).
- The presence of a gradient introduced an additional effective force in the deterministic part of the equation of motion. It consisted of a component 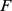 pointing in gradient direction and a component 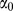 pointing in the direction of propagation, see Fig. 3 and Eqs. 2 and 3. The stochastic part did not depend on direction.
- With increasing gradient steepness, both 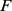 and 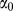 went through a maximum, coinciding with a peak in the chemotactic index, see Figs. 5 and 1D. The damping coefficient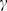, on the other hand, did not show any gradient dependence.
- The parameters 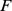 and 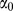 were related to distinct types of cell motion. While high values of 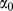 reflected a large mean speed, a large value 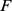 corresponded to increased chemotactic efficiency. This was demonstrated by considering subpopulations of different speed and chemotactic index, see Fig. 6. Moreover, faster cells showed a higher noise level.

Note that in general, chemotactic movement will depend on both the chemoattractant gradient and the average ambient chemoattractant concentration (the so-called midpoint concentration). In the data presented here, the cells are exposed to a constant gradient, while the midpoint concentration increases when the cells are moving up the gradient. Our data thus presents a global average over a range of midpoint concentrations for each gradient investigated. In order to also resolve the dependence on the midpoint concentration, the cell trajectories would need to be divided into small intervals along the gradient direction to perform the stochastic data analysis within each interval, i.e., for each midpoint concentration, separately. However, even though we have collected a substantial amount of data, much larger data sets would be required in order to obatin statistically meaningful results from this type of analysis. This is primarily because the stochastic data analysis requires an additional division of the data also according to angle and speed. In Fig. S9 of the electronic supplementary material, we present a coarse grained version of this analysis, where the gradient region has been divided into two intervals. No clear trend was found for the speed and the chemotactic index.

In previous studies, it has been shown for human dermal fibroblasts that the damping parameter 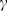 depends on cell-substrate interactions. In particular, different surface coatings induced strong changes in the value of 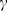 for the same cell type [15]. As we did not change the surface properties in our present study, the observation of a constant 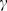 for different gradients suggests that also in the case of *Dictyostelium* chemotaxis, the parameter 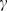 might be mostly reflecting the cell-surface interactions. We will test this conjecture in future studies by exploring the chemotactic performance of *Dictyostelium* cells on surfaces with different coatings.

Furthermore, the noise term was found to be independent of the external gradient. Together with our earlier observation that the stochastic components of non-directional motility are not affected by development or ambient cAMP [19], this supports the hypothesis that cell motion is influenced by an independent random process. We assume that this random component is related to pseudopod formation. This is motivated by earlier results demonstrating that pseudopod formation is a random event [28]. In agreement with our observations, it was reported that the temporal dynamics of this process (frequency of pseudopod formation) is not affected by the presence of a gradient, which only imposes a spatial bias (preferred direction of pseudopod formation) [29]. Note that we image with a time interval of 40 sec, while pseudopods are formed in cycles of about 10 sec. We thus do not resolve the entire process of pseudopod formation but rather sample the cell shape at independent time points that are not correlated with respect to the time scale of pseudopod formation.

Our model can be considered as a description that captures the behavior of a representative, average individual from the chemotactic cell population. The mean values and fluctuations of various motion parameters are correctly captured for this average chemotactic cell. However, by subdividing the cells into subpopulations of different motility and chemotactic performance, we demonstrated that a considerable cell to cell variability exists and that the parameters of the Langevin equation are different for each subpopulation.

Also the form of the stochastic part is influenced by the heterogeneity of a typical cell population. While the slopes 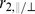 take positive values when determined for the entire population of cells, we found a slope close to zero in the subpopulations of fast moving cells. Also, the offsets 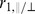 are larger for subpopulations of fast cells than for slow cells. This indicated that the multiplicative noise observed for the whole cell population is a superposition of noise contributions that may have a different character at the level of the individual cells.

We can also relate the multiplicative noise in our Langevin equation to the stochastic processes that occur in the cell during gradient sensing. To the best of our knowledge, the only purely stochastic model of gradient sensing was presented by Gamba et al. [30], and extensively characterized in subsequent publications, see for example [31]. When simplified, the original model can be reduced to a reaction-diffusion system, where reactions are nonlinear. In recent years, many such nonlinear models have been proposed to describe directional processes in chemotactic cells, for examples see [32]–[34]. This nonlinearity leads to multiplicative noise [31], [35]. The multiplicative noise observed at the motility level could therefore be a direct consequence of the nonlinearity of the gradient-sensing mechanism.

In a recent study of *Dictyostelium* chemotaxis in exponential gradients, the average cell speed did not depend on the direction of propagation. This led to a description based on a Langevin equation for the angle of propagation only [36]. On the contrary, non-uniform distributions for both the angle of propagation and the speed were observed in our study with linear chemoattractant gradients. Here, both distributions show a maximum in gradient direction, see Fig. 2D and E. Thus, in this case, directional motion results from two combined effects, (a) cells are more likely to move in gradient direction and (b) their speed is larger when moving in gradient direction. This is in agreement with work reported in [37].

In future work, we will apply our analysis to mutant cell lines that carry deficiencies in various components of the chemotactic signaling pathway. The objective is to relate the specific parameters of our stochastic description to the individual molecular players in a chemotaxing cell. Such relations between microscopic molecular components and macroscopic dynamical observables are an essential building block for a comprehensive model of eukaryotic chemotaxis, the central aim of this field.

## Materials and Methods

### Cell Culture

All experiments were performed with *Dictyostelium* AX3 wild type cells, kindly provided by Günther Gerisch (MPI for Biochemistry, Martinsried, Germany). Cells were grown in HL5 medium (7 g/L yeast extract, 14 g/L peptone, 0.5 g/L potassium dihydrogen phosphate, 0.5 g/L disodium hydrogen phosphate, 13.5 g/L glucose, ForMedium Ltd., UK). The culture was renewed from frozen stock every four weeks. Cells were starved in shaking suspension of phosphate buffer (pH 6.0, 15 mM KH
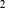
PO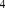, 1 mM Na
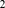
HPO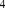) at a density of 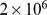 cells/mL for 5:30 hours. After one hour of starvation, the cells were exposed to periodic pulses of cAMP for the remaining time of starvation. The pulses had a concentration of 50nM and were delivered with a period of 6 minutes.

### Microfluidics

The experiments were performed in a microfluidic gradient mixer, in which stable gradient profiles could be established over a region of 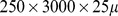
m in size. The design of the gradient mixer was an adapted version of the pyramidal network pioneered by Jeon et al. [24] that allowed for the generation of linear concentration profiles between two arbitrarily chosen input concentrations. The layout of the gradient mixer is displayed in Fig. 1B, for a detailed description, see Ref. [6]. The microfluidic device was built by standard microfabrication procedures, generally referred to as soft lithography. Based on photolithographic techniques, a Si wafer (Wafer World Inc.) was spin coated with photoresist (SU-8 50, Micro Resist Technology) in a clean room environment. Patterning by UV light exposure and chemical development produced a ‘master wafer’ that carried a relief of the desired microstructure. This was used in a polymer molding step to cast the microstructure into premixed polydimethylsiloxane (PDMS; Sylgard 184, Dow Corning). After 1h of curing at 65°C, the PDMS was removed from the master wafer and fluid inlets and outlets were punched through the polymer using a sharpened syringe tip (19 gauge×1 in., McMaster). The molded PDMS block was then sealed from below with a glass cover slip (24×60 mm, No. 1, Gerhard Menzel Glasbearbeitungswerk GmbH & Co. KG). Bonding between the PDMS and the glass was achieved by a preceding treatment of all surfaces in an air plasma (PDC-002, Harrick Plsama) for 3 min. 500 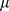
L gastight glass syringes (1750 TTLX, Hamilton Bonaduz AG) were mounted on a precision syringe pump (PHD 2000, Harvard Apparatus Inc.) and connected to the microfluidic device with Teflon tubing (39241, Novodirect GmbH) to ensure a constant supply of liquids. A detailed review of soft lithography can be found in Ref. [38].

We have performed control experiments with cells migrating in the microfluidic device under identical flow conditions but in absence of a chemoattractant gradient. No effect of the fluid flow on the cell motion could be detected. In particular, the histograms of the x- and y-components of the velocity were symmetric and superposed almost perfectly. See Fig. S8 of the electronic supplementary material, where examples of these histograms are displayed. Note also, that the parameters were chosen such that flow-induced distortions of the chemical gradient signal in the vicinity of the cells were kept minimal [39].

### Microscopy and Image Processing

Cell tracks were recorded on a Deltavision RT microscope imaging system (Applied Precision, Inc.). Pictures were taken with a 10x plan apochromat (UPLSAPO, Olympus) objective every 40 seconds during 50 minutes using a Photometrics CoolSnap CCD camera (Princeton Instruments, Inc.) at a resolution of 1024x1024 pixels. Differential interference contrast (DIC) was used to enhance cell contour visualization. About 120 cell tracks were recorded for each experiment. The cell contours were automatically detected using a method inspired by Kam [40] that was implemented in a MATLAB program (Mathworks). The cell centroid was then computed on the basis of the cell contour. The error in the contour finding algorithm leads to an error in the position of the cell centroid. The time interval of 40 sec between subsequent frames was chosen such that, given the average cell speed, a cell travels a distance that is larger than the error in the cell centroid position during one time step. See Ref. [41] for details of this method. Between subsequent frames, the centroids of the cell contours were tracked using a custom-made MATLAB program based on the tracking algorithm of Crocker and Grier [42]. The first 10 minutes of data were systematically discarded, as they corresponded to the time where the concentration gradient was not yet stationary.

### Stochastic Data Analysis

For each cell track, the velocity and acceleration of the cell was calculated at each point by finite differences from the cell positions. The deterministic and the stochastic parts of motion were separated according to equation 1. We determined the functions 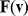 and 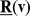 in equation 1 from experimental data, using conditional averages as pioneered by Siegert et al. [26]. We checked that the Chapman-Kolmogorov condition was verified [26]. We expressed velocity and acceleration in a moving coordinate frame where the two unit vectors 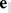 and 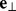 point parallel and perpendicularly to the current velocity of the cell, respectively (see Fig. 1A). We denote the speed by 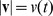 and the angle of propagation with respect to the gradient direction by 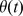. To perform conditional averaging, we divided the range of cell speeds into 20 bins of equal size 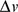. The range of propagation angles was divided into intervals of 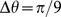. We can then find 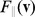 by approximating.
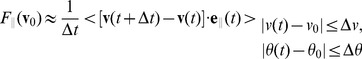
(4)where 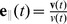, 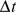 is the (discrete) experimental time interval, and 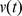 is within 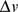 of 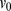, while the angle 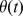 is within 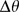 of 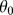 [26], [27], [43]. The perpendicular component 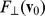 is found in a similar way, by replacing 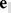 in equation 4 by 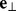. The noise terms can be approximated by



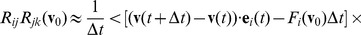


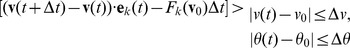
(5)


The cross-correlation of the acceleration components was found to be neglectible as compared to the autocorrelation of each individual component. We could therefore conclude that there were no mixed stochastic terms, so that the stochastic contributions in the parallel and perpendicular directions could be computed according to
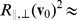
(6)

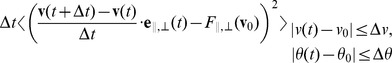



Furthermore, because no angle dependence was found in either of the stochastic components, we re-evaluated them without angular binning and fitted the results by a first order polynomial 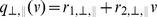.

### Simulations

An Euler-Maruyama scheme was used to simulate the equations 2 and 3 with the parameters obtained from our experimental data [44]. The time step of the simulations was the same as the time step used for the conditional averaging (40 seconds). We simulated 100 tracks, each track being 200 points long (33 minutes).

## Supporting Information

Figure S1
**Directional dependence of the friction coefficient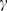.** The friction coefficient is shown as a function of 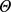. It is independent of the cell’s direction with respect to the gradient.(TIFF)

Figure S2
**Effective force term 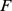.** To establish a relation between the amplitudes 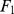 and 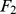 of the angle dependent contributions, we display 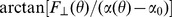 as a function of 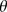. It can be seen that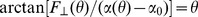, demonstrating that 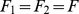.(TIFF)

Figure S3
**Noise perpendicular to the direction of motion.** (A) Stochastic component of the perpendicular acceleration. Black dots show the experimental data, the red lines show a linear fit 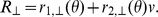 (B, C) 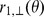 and 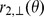 do not depend on 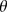. The red lines show constant fits.(TIFF)

Figure S4
**Gradient dependence of the stochastic part.** Constants 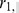, 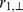, 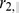 and 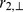 retrieved from linear fitting of the stochastic part for different gradients (red: perpendicular, black: parallel). As in Fig. 1D of the main text, the data point displayed at very low gradient values (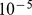
nM/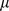
m) corresponds to an experiment where no gradient of cAMP was applied.(TIFF)

Figure S5
**Stochastic part of subpopulations.** Constants 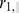, 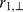, 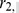 and 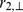 retrieved from linear fitting of the stochastic parts of different subpopulations.(TIFF)

Figure S6 **Conditional averaging for the non-directional case.** Left: The parallel acceleration (red datapoints) can be fitted by a quadratic term (red line) while the perpendicular acceleration is zero and independent of the velocity (green datapoints and constant fit). Right: The stochastic components in the parallel (red) and perpendicular direction (green) can be fitted by a first-order polynomial.
(TIFF)

Figure S7
**Stochastic components of the Langevin equation.** Stochastic component of parallel acceleration, for (a) 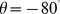 and (b) 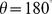. (c) Stochastic component of parallel acceleration, averaged over all angles. Black dots show the experimental data, the red line shows a linear fit 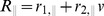.(TIFF)

Figure S8 **Testing the influence of flow forces.** Histograms of the velocity components in x- and y-direction in absence of a chemoattractant gradient (the x-direction corresponding to the direction of fluid flow). Both histograms superpose closely, indicating that the fluid flow does not induce any preferred direction of cell motion.
(TIFF)

Figure S9
**Testing the influence of cell position in the chamber.** Scatter plot displaying each cell according to its mean speed and chemotactic index as a dot in the (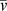,CI)-plane. Black dots denote cells in the lower half of the microfluidic device (i.e., lower half of the gradient), red dots mark cells in the upper half. On average, the black cells move faster (8.5 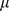
m/min) than the red cells (7.6 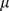
m/min) but their chemotactic index is lower (0.25) than the chemotactic index of the red cells (0.36).(TIFF)
