# Supplementary figures and images for: A Stochastic Description of Dictyostelium Chemotaxis
Source: PLoS One. 2012 May 25;7(5):e37213. doi: 10.1371/journal.pone.0037213 (PMC3360683; doi:10.1371/journal.pone.0037213)

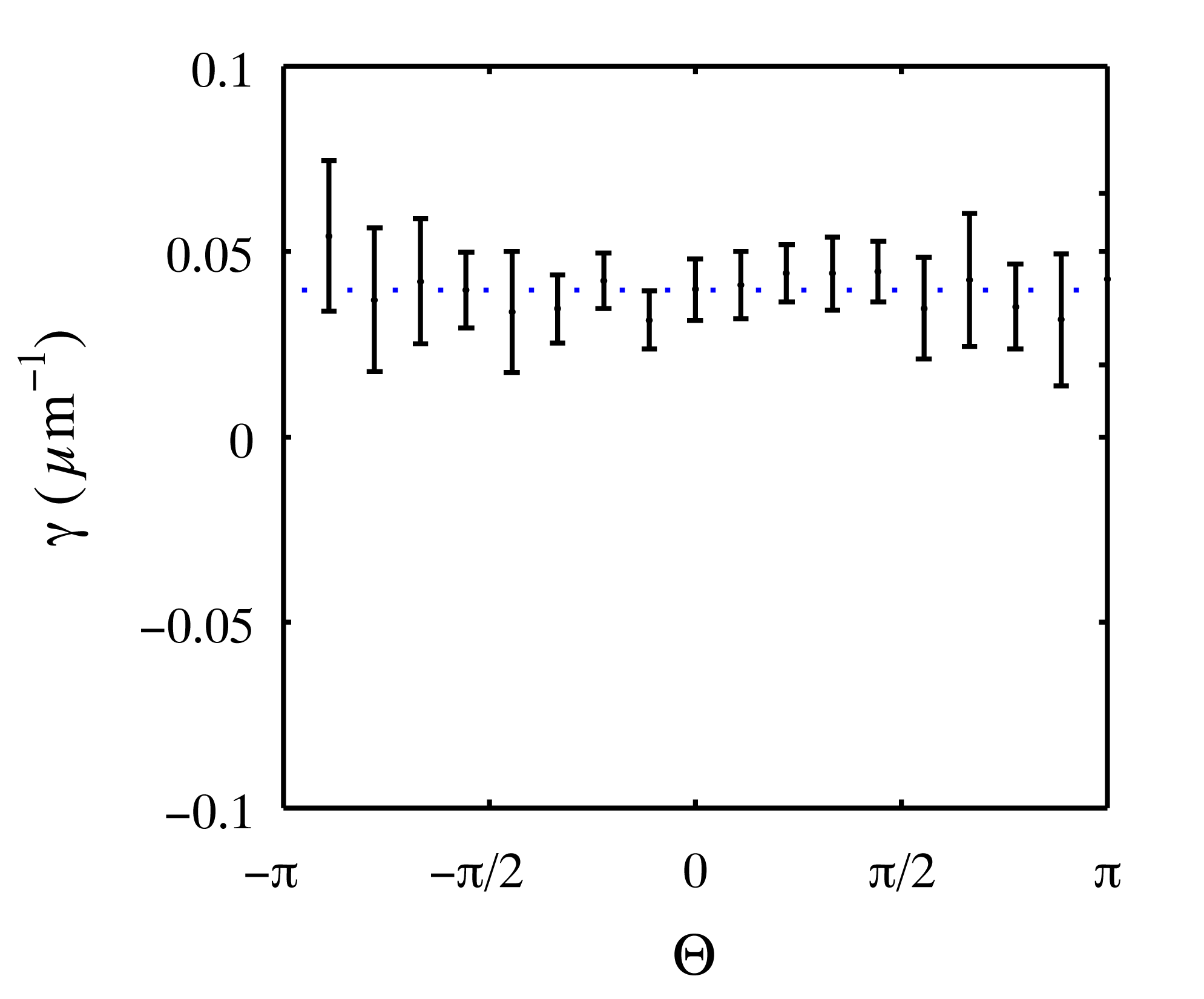

Supplement: Figure S1 — Directional dependence of the friction coefficient. The friction coefficient is shown as a function of . It is independent of the cell’s direction with respect to the gradient. (TIFF) [file pone.0037213.s001.tiff]

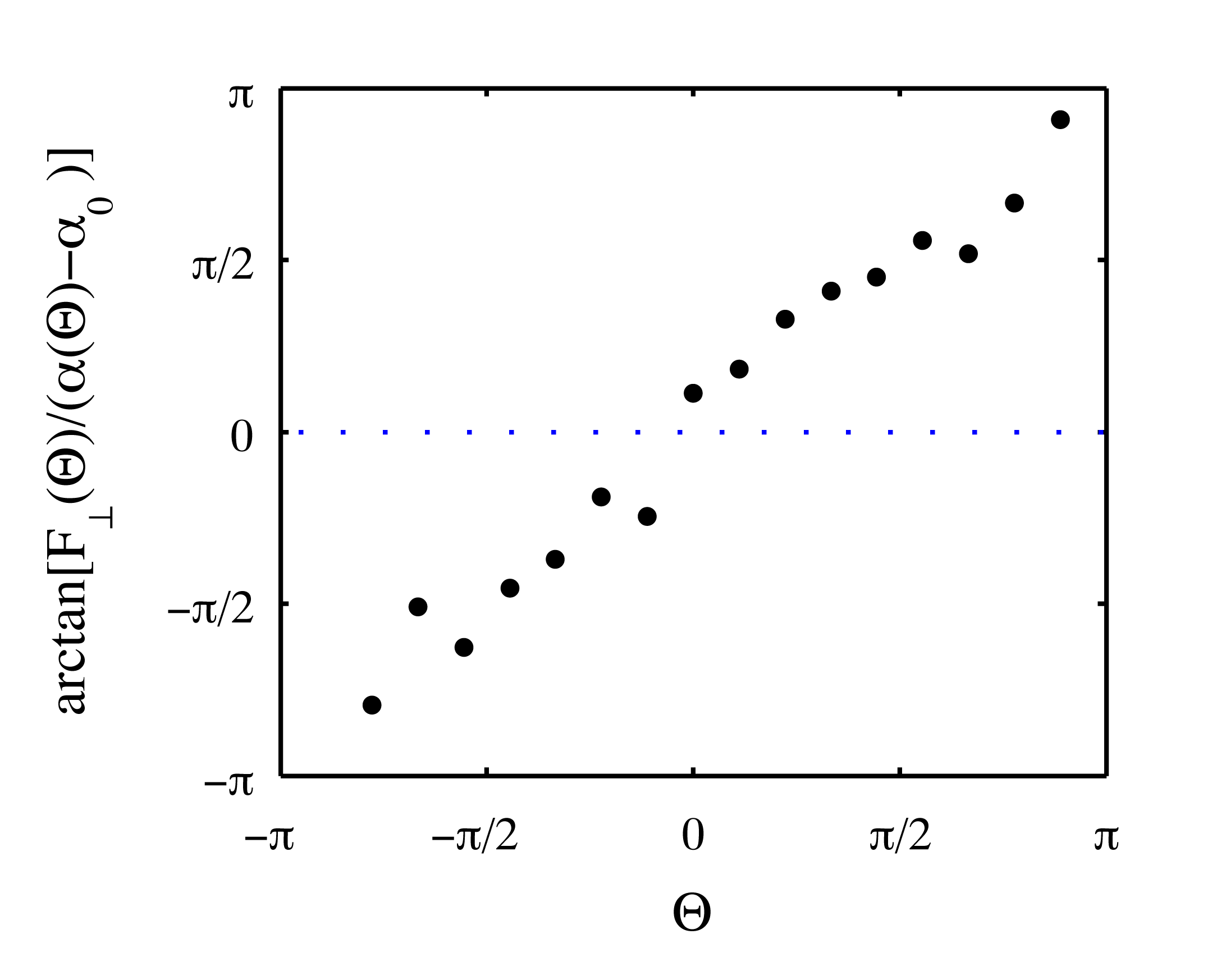

Supplement: Figure S2 — Effective force term . To establish a relation between the amplitudes and of the angle dependent contributions, we display as a function of . It can be seen that, demonstrating that . (TIFF) [file pone.0037213.s002.tiff]

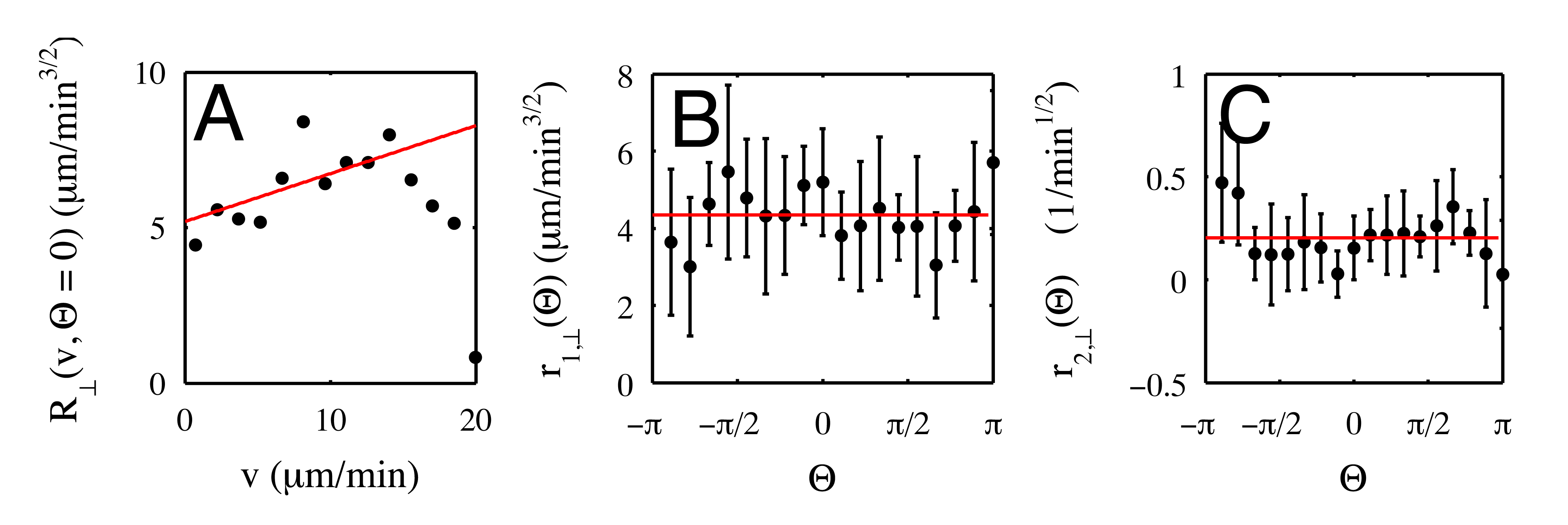

Supplement: Figure S3 — Noise perpendicular to the direction of motion. (A) Stochastic component of the perpendicular acceleration. Black dots show the experimental data, the red lines show a linear fit (B, C) and do not depend on . The red lines show constant fits. (TIFF) [file pone.0037213.s003.tiff]

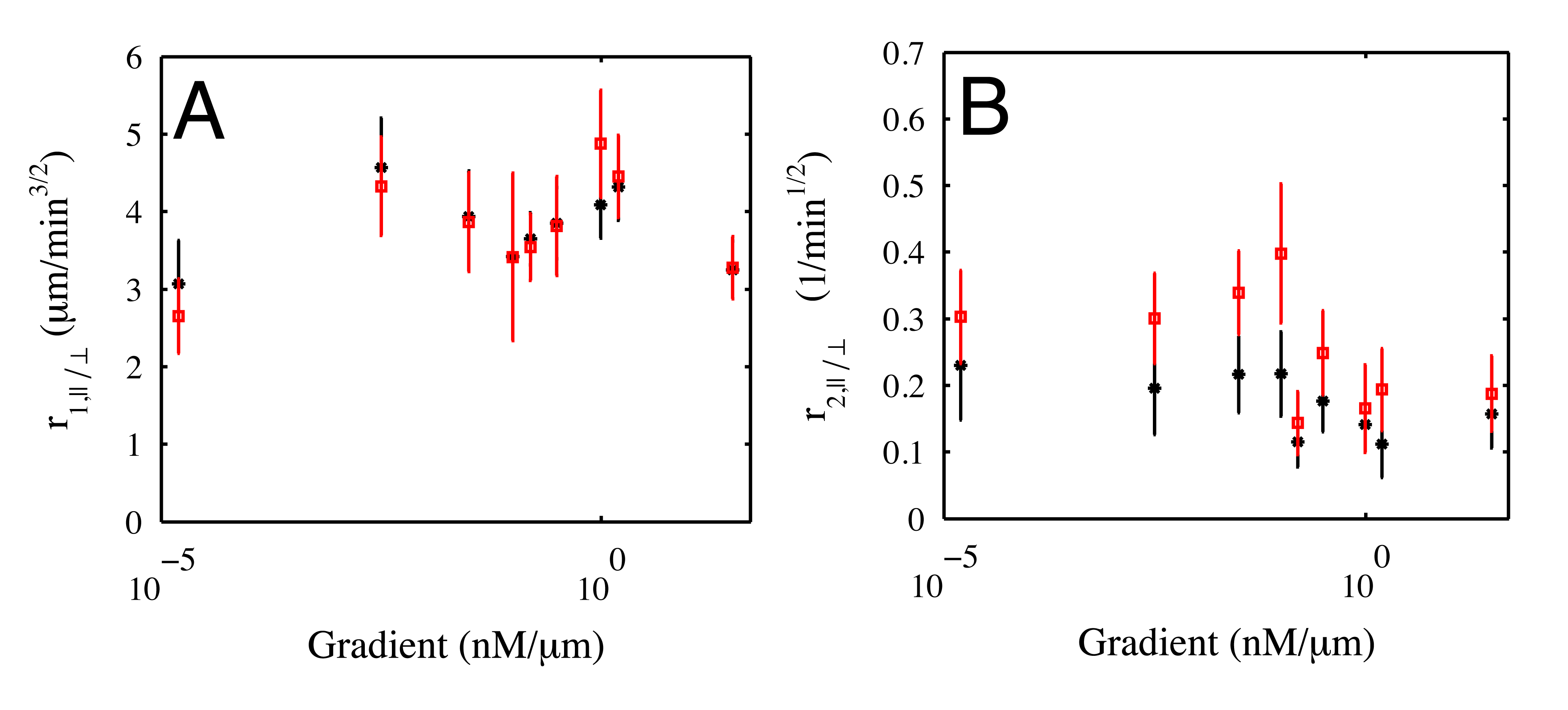

Supplement: Figure S4 — Gradient dependence of the stochastic part. Constants , , and retrieved from linear fitting of the stochastic part for different gradients (red: perpendicular, black: parallel). As in Fig. 1D of the main text, the data point displayed at very low gradient values (nM/m) corresponds to an experiment where no gradient of cAMP was applied. (TIFF) [file pone.0037213.s004.tiff]

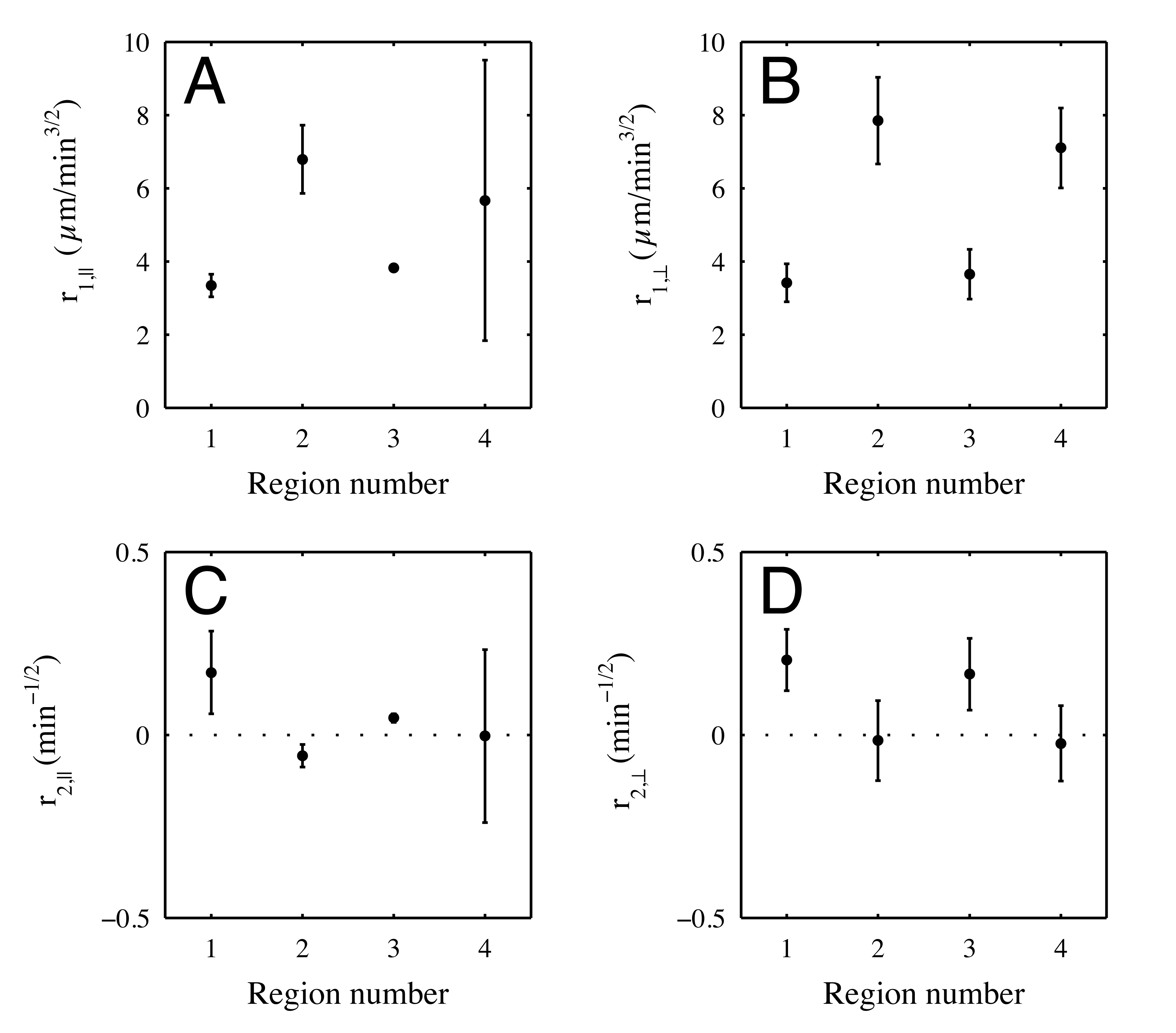

Supplement: Figure S5 — Stochastic part of subpopulations. Constants , , and retrieved from linear fitting of the stochastic parts of different subpopulations. (TIFF) [file pone.0037213.s005.tiff]

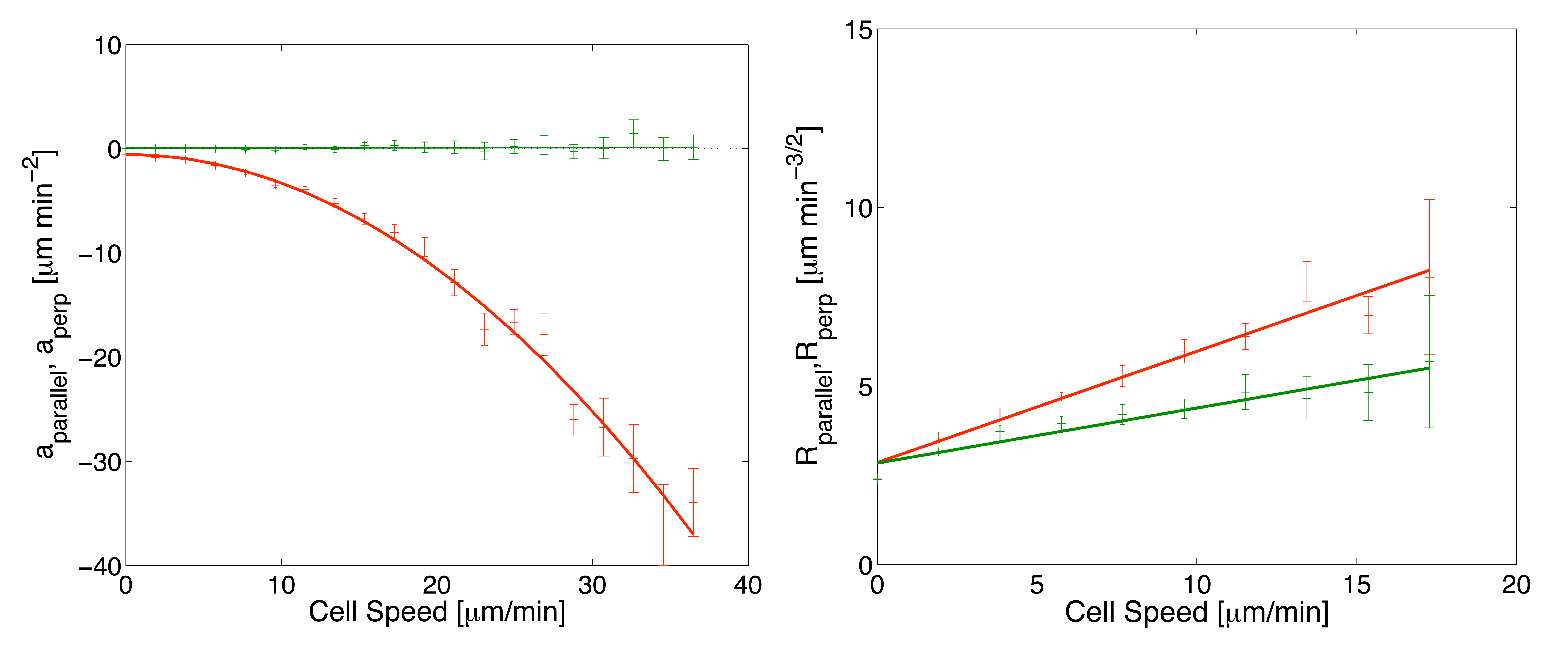

Supplement: Figure S6 — Conditional averaging for the non-directional case. Left: The parallel acceleration (red datapoints) can be fitted by a quadratic term (red line) while the perpendicular acceleration is zero and independent of the velocity (green datapoints and constant fit). Right: The stochastic components in the parallel (red) and perpendicular direction (green) can be fitted by a first-order polynomial. (TIFF) [file pone.0037213.s006.tiff]

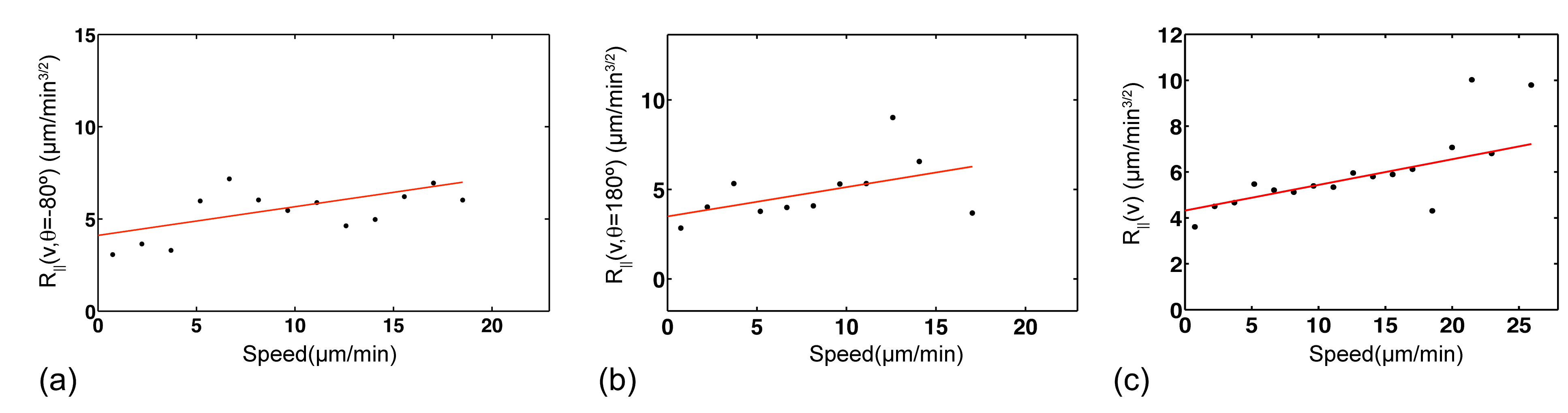

Supplement: Figure S7 — Stochastic components of the Langevin equation. Stochastic component of parallel acceleration, for (a) and (b) . (c) Stochastic component of parallel acceleration, averaged over all angles. Black dots show the experimental data, the red line shows a linear fit . (TIFF) [file pone.0037213.s007.tiff]

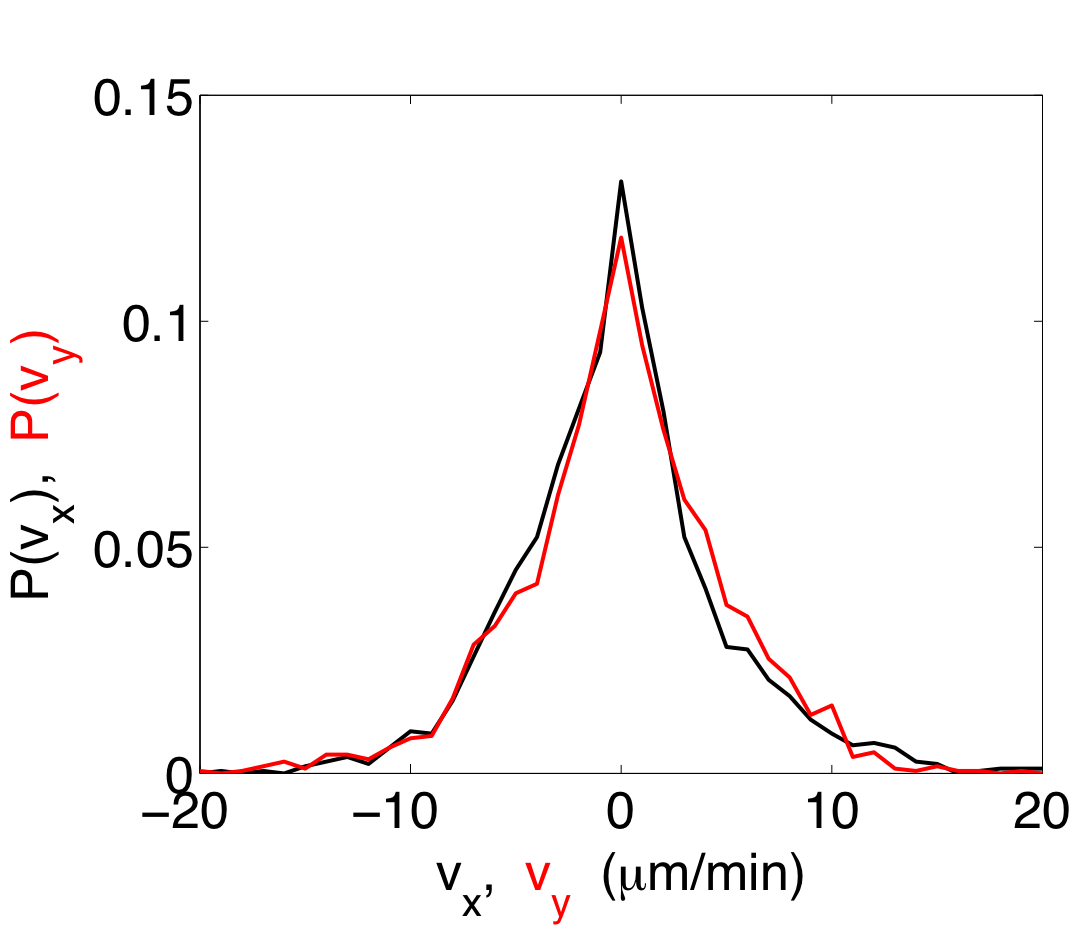

Supplement: Figure S8 — Testing the influence of flow forces. Histograms of the velocity components in x- and y-direction in absence of a chemoattractant gradient (the x-direction corresponding to the direction of fluid flow). Both histograms superpose closely, indicating that the fluid flow does not induce any preferred direction of cell motion. (TIFF) [file pone.0037213.s008.tiff]

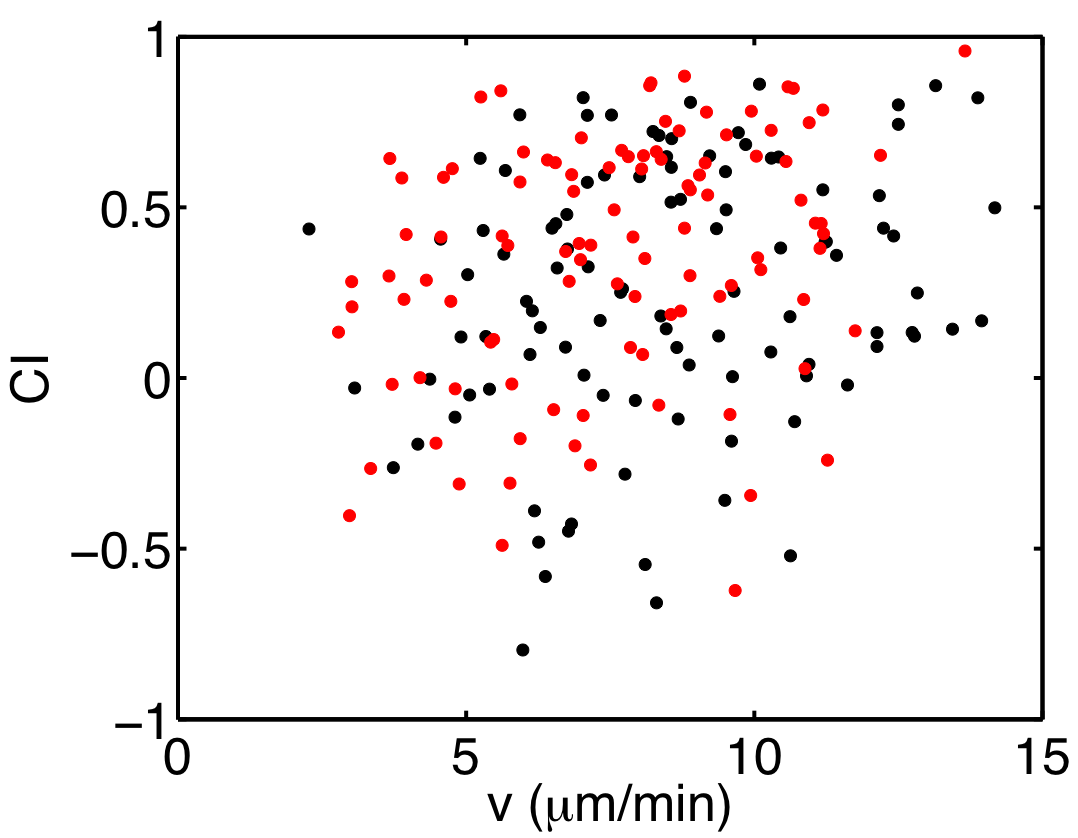

Supplement: Figure S9 — Testing the influence of cell position in the chamber. Scatter plot displaying each cell according to its mean speed and chemotactic index as a dot in the (,CI)-plane. Black dots denote cells in the lower half of the microfluidic device (i.e., lower half of the gradient), red dots mark cells in the upper half. On average, the black cells move faster (8.5 m/min) than the red cells (7.6 m/min) but their chemotactic index is lower (0.25) than the chemotactic index of the red cells (0.36). (TIFF) [file pone.0037213.s009.tiff]
